# Data-Independent Acquisition Proteomics Reveals Long-Term Biomarkers in the Serum of C57BL/6J Mice Following Local High-Dose Heart Irradiation

**DOI:** 10.3389/fpubh.2021.678856

**Published:** 2021-07-02

**Authors:** Omid Azimzadeh, Christine von Toerne, Vikram Subramanian, Wolfgang Sievert, Gabriele Multhoff, Michael J. Atkinson, Soile Tapio

**Affiliations:** ^1^Institute of Radiation Biology, Helmholtz Zentrum München - German Research Center for Environmental Health, Neuherberg, Germany; ^2^Section Radiation Biology, Federal Office for Radiation Protection, Oberschleissheim, Germany; ^3^Research Unit Protein Science, Helmholtz Zentrum München - German Research Center for Environmental Health, Munich, Germany; ^4^Department of Radiation Oncology, Center for Translational Cancer Research (TranslaTUM), Campus Klinikum rechts der Isar, Technical University of Munich, Munich, Germany; ^5^Radiation Biology, Technical University of Munich, Munich, Germany; ^6^Institute for Biological and Medical Imaging, Helmholtz Zentrum München - German Research Center for Environmental Health, Neuherberg, Germany

**Keywords:** radiation therapy, proteomics, data-independent acquisition, inflammation, ionizing radiation, biomarker, radiation-induced heart disease, cardiac lipid metabolism

## Abstract

**Background and Purpose:** Cardiotoxicity is a well-known adverse effect of radiation therapy. Measurable abnormalities in the heart function indicate advanced and often irreversible heart damage. Therefore, early detection of cardiac toxicity is necessary to delay and alleviate the development of the disease. The present study investigated long-term serum proteome alterations following local heart irradiation using a mouse model with the aim to detect biomarkers of radiation-induced cardiac toxicity.

**Materials and Methods:** Serum samples from C57BL/6J mice were collected 20 weeks after local heart irradiation with 8 or 16 Gy X-ray; the controls were sham-irradiated. The samples were analyzed by quantitative proteomics based on data-independent acquisition mass spectrometry. The proteomics data were further investigated using bioinformatics and ELISA.

**Results:** The analysis showed radiation-induced changes in the level of several serum proteins involved in the acute phase response, inflammation, and cholesterol metabolism. We found significantly enhanced expression of proinflammatory cytokines (TNF-α, TGF-β, IL-1, and IL-6) in the serum of the irradiated mice. The level of free fatty acids, total cholesterol, low-density lipoprotein (LDL), and oxidized LDL was increased, whereas that of high-density lipoprotein was decreased by irradiation.

**Conclusions:** This study provides information on systemic effects of heart irradiation. It elucidates a radiation fingerprint in the serum that may be used to elucidate adverse cardiac effects after radiation therapy.

## Introduction

It is, nowadays, commonly acknowledged that the exposure of the heart to ionizing radiation, as in radiation therapy for breast cancer, Hodgkin's disease, or other cancers of the chest, increases the risk of heart disease (1, 2). This has become a growing problem with the advancements in cancer therapy that have successfully reduced both mortality rates and the recurrence, expanding the life expectancy of the survivors (3, 4).

Manifestations of radiation-induced heart disease include pericarditis, pericardial fibrosis, diffuse myocardial fibrosis, coronary artery disease, microvascular damage, and stenosis of the valves (5, 6). Considering causal biological processes in the development of the disease, persistent inflammation and oxidative stress, fibrosis, and pre-mature endothelial senescence are thought to be salient (7–9). Recently, the role of mitochondrial dysfunction and related metabolic perturbations has become more and more evident (10–12).

Detecting cardiac toxicity by assessing left ventricular function often requires a large amount of myocardial damage, characteristic of irreversible heart injury (13, 14). There is increasing emphasis on the use of biomarkers to detect cardiotoxicity at a stage before it becomes irreversible.

The most important blood biomarkers of heart injury are cardiac troponins T (cTnT) and I (cTnI), heart proteins controlling the calcium-mediated interaction between actin and myosin filaments (15). While cTnT is expressed to a small extent in skeletal muscle, cTnI has been found only in the myocardium. A previous study by Skyttä et al. showed that cTnT levels increased during adjuvant whole-breast radiation therapy in one out of five patients. Moreover, the increase in cTnT release was positively associated with cardiac radiation dose and with minor changes in the left ventricular diastolic function (16). A sustained irreversible leakage of cardiac troponins to the blood stream is due to the degradation of the myofibrils after heart damage (17).

Since irradiation tends to stimulate inflammatory processes, C-reactive protein (CPR), an acute phase protein, could be an additional potential predictive marker of cardiotoxicity after irradiation. Increased CRP level was associated with the severity of radiation-induced cardiomyopathy after radiation therapy of lung or breast cancer (18). We have shown elevated levels of inflammatory cytokines such as interleukin (IL)-1, IL-6, and tissue necrosis factor alpha (TNF-α) in serum after local cardiac irradiation in mice (19), but data on their role in the prediction of myocardial changes in clinical trials are lacking to date (20, 21).

Although cardiac troponins and CRP are established sensitive biomarkers of myocardial injury and inflammation, respectively, there is no specificity for radiation-associated heart disease. In fact, there are no biomarkers available to identify radiotherapy patients who are in the process of developing radiation-associated heart disease, although the current data suggest that certain blood biomarkers may be associated with myocardial dysfunction (20, 22, 23).

Proteomics represents a promising global technology to discover new types of biomarkers for radiation-induced cardiac injury (11, 24). However, identification and quantification of serum/plasma proteins remains an analytical challenge, mainly due to the dominance of albumins and immunoglobulins and the high dynamic range of protein abundances (25). This is particularly true for disease-specific biomarkers that are mainly low-abundance proteins (26, 27). The newly established quantitative proteomics technology based on the data-independent acquisition (DIA) mass spectrometry (MS) was introduced recently to overcome the limitation of previous approaches (26). The aim of this study was to identify biomarkers of cardiac toxicity in the serum proteome of mice after local heart irradiation by using DIA-MS.

## Materials and Methods

### Irradiation and Sample Preparation

Local heart irradiation was carried out on male C57BL/6J mice at the age of 8 weeks as previously described (28). Briefly, mice were irradiated with a single X-ray dose of 8 or 16 Gy locally to the heart (200 kV, 10 mA) (Gulmay, West Byfleet, UK). The age-matched control mice were sham irradiated. Mice were not anesthetized during irradiation but were held in a prone position in restraining jigs with the thorax fixed using adjustable hinges. The position and field size (9 × 13 mm^2^) of the heart was determined by pilot studies using soft X-rays; the rest of the body was shielded with a 2-mm-thick lead plate. With this beam size, 40% of the lung volume receives, by necessity, the full heart dose (29). Blood samples were collected from all mice by cardiac puncture after animals were sacrificed 20 weeks post-radiation. The serum was isolated and kept at −80°C for further analyses. All animal experiments were approved and licensed under Bavarian federal law (Certificate No. AZ 55.2-1-54-2532-114-2014). Altogether, 15 mice were used in this study, with five mice in each group.

### Proteome Profiling

Serum protein concentrations were determined by Bradford assay, and 50 μg per sample was prepared using PreOmics' iST Kit (Preomics GmbH, Martinsried, Germany) according to manufacturers' specifications. After drying, the peptides were resuspended in 2% acetonitrile (ACN) and 0.5% trifluoroacetic acid. The HRM Calibration Kit (Biognosys, Schlieren, Switzerland) was added to all of the samples according to the manufacturer's instructions.

The MS data were acquired in DIA mode on a Q Exactive HF mass spectrometer (Thermo Fisher Scientific Inc., Waltham, MA, USA). Samples were automatically loaded to the online coupled RSLC (Ultimate 3000, Thermo Fisher Scientific Inc.) HPLC system. A Nano-Trap column was used (300-μm inner diameter (ID) × 5 mm, packed with Acclaim PepMap100 C18, 5 μm, 100 Å from LC Packings, Sunnyvale, CA, USA, before separation by reversed-phase chromatography (Acquity UPLC M-Class HSS T3 Column 75 μm ID × 250 mm, 1.8 μm from Waters, Eschborn, Germany) at 40°C. Peptides were eluted from the column at 250 nl/min using increasing ACN concentration in 0.1% formic acid from 3 to 40% over a 45-min gradient.

The DIA method consisted of a survey scan from 300 to 1,500 m/z at 120,000 resolution and an automatic gain control (AGC) target of 3e6 or 120-ms maximum injection time. Fragmentation was performed via higher-energy collisional dissociation with a target value of 3e6 ions determined with predictive AGC. Precursor peptides were isolated with 17 variable windows spanning from 300 to 1,500 m/z at 30,000 resolution with an AGC target of 3e6 and automatic injection time. The normalized collision energy was 28, and the spectra were recorded in profile type.

Selected LC-MS/MS data encompassing 164 raw files were analyzed using Proteome Discoverer (Version 2.1, Thermo Fisher Scientific Inc.) using Byonic (Version 2.0, Proteinmetrics, San Carlos, CA, USA) search engine node maintaining 1% peptide and protein FDR threshold. The peptide spectral library was generated in Spectronaut (Version 10, Biognosys, Schlieren, Switzerland) with default settings using the Proteome Discoverer result file. Spectronaut was equipped with the Swiss-Prot mouse database (Release 2017.02, 16,869 sequences, www.uniprot.org) with a few spiked proteins (e.g., Biognosys iRT peptide sequences). The final spectral library generated in Spectronaut contained 10,525 protein groups and 322,041 peptide precursors. The DIA-MS data were analyzed using the Spectronaut 10 software applying default settings with the exception: quantification was limited to proteotypic peptides, data filtering was set to *Q*-value 25% percentile, summing-up peptide abundances. For this study, the proteins with a *q*-value <0.05 were considered as significantly differentially expressed.

Additional differential abundance testing was performed in Spectronaut as unpaired ratio based *t*-test on peptide level to identify the candidates' differential between the experimental groups (i) sham irradiation, (ii) 8-Gy irradiation, or (iii) 16-Gy irradiation.

### Interaction and Signaling Network Analysis

The analyses of protein–protein interaction and signaling networks were performed by the software tools INGENUITY Pathway Analysis (IPA) (Qiagen, Inc., Hilden, Germany, https://www.qiagenbioinformatics.com/products/ingenuity-pathway-analysis) (30).

### Serum Inflammatory Molecule Analysis

The expression levels of different mediators including TNF-α, TGF-β, monocyte chemoattractant protein 1 (MCP1), IL-1 α, IL-1 β, IL-6, IL-10, IL-12, interferon (IFN) gamma, granulocyte-colony stimulating factor (G-CSF), and granulocyte–macrophage colony-stimulating factor (GM-CSF) were measured using ELISA strip colorimetric kits #EA-1401, # EA-1051, and # EA-1131 (Signosis, Inc., Santa Clara, CA, USA) according to the manufacturer's instructions.

### Serum Lipid Profiling

The levels of circulating free fatty acids (ab65341), triglyceride (ab65336), total cholesterol, low-density lipoprotein (LDL), and high-density lipoprotein (HDL) (ab65390), all from Abcam, Cambridge, MA, USA, and oxidized low-density lipoprotein (oxLDL) (MBS2512757, MyBioSource, San Diego, CA, USA) were measured according to the manufacturer's instructions.

### Statistical Analysis

The 3D principal component analysis (PCA) was performed by R (4.0.5) (https://www.R-project.org/) and the hierarchical clustering using the Heatmapper web server (http://www.heatmapper.ca/) (31). Student's *t*-test (unpaired) was used for proteomics and ELISA comparisons. The error bars were calculated as the standard error of the mean (SEM).

### Data Availability

The mass spectrometry proteomics data have been deposited to the ProteomeXchange Consortium via the PRIDE (32) partner repository with the dataset identifier PXD024446.

## Results

### Serum Proteome Alterations Following Local Heart Irradiation

The serum proteome of mice was analyzed 20 weeks in sham-irradiated and irradiated (8 and 16 Gy) mice using DIA-MS. The analysis identified and quantified 499 proteins (Supplementary Table 1). Among the quantified proteins, the expression of 42 and 59 proteins was significantly changed (*q*-value <0.05, identification by at least two unique peptides) at 8 and 16 Gy, respectively, suggesting a dose-dependent increase in the number of significantly deregulated proteins (Table 1). The majority of these proteins (76% at 8 Gy and 83% at 16 Gy) have been previously annotated as serum proteins based on the Plasma Proteome Database (PPD) (http://plasmaproteomedatabase.org/index.html).

**Table 1:** Significantly deregulated serum proteins in heart-irradiated mice.

**#**	**Protein accession**	**Protein ID**	**Protein description**	**Total unique peptides**	**Ratio 8/0 Gy**	**Ratio 16/0 Gy**
1	P48410	ABCD1	ATP-binding cassette subfamily D member 1	20		0.712
**2**	**P29699**	**AHSG**	**Alpha-2-HS-glycoprotein**	**12**	**0.989**	**1.043**
3	P05064	ALDOA	Fructose-bisphosphate aldolase A	48	0.435	
4	Q91Y97	ALDOB	Fructose-bisphosphate aldolase B	5	0.476	
5	P12246	APCS	Serum amyloid P-component	3	2.375	
6	P09813	APOA2	Apolipoprotein A-II	3	0.812	
**7**	**P06728**	**APOA4**	**Apolipoprotein A-IV**	**28**	**0.891**	**0.993**
**8**	**E9Q414**	**APOB**	**Apolipoprotein B-100**	**16**	**1.301**	**1.271**
9	P34928	APOC1	Apolipoprotein C-I	3	0.801	
10	P08226	APOE	Apolipoprotein E	24		1.340
11	Q02105	C1QC	Complement C1q subcomponent subunit C	5		0.857
12	Q80X80	C2CD2L	C2 domain-containing protein 2-like	31		0.039
**13**	**P01027**	**C3**	**Complement C3**	**99**	**1.153**	**0.895**
**14**	**P01029**	**C4B**	**Complement C4-B**	**45**	**1.452**	**1.151**
15	P06684	C5	Complement C5	20	1.134	
16	P55284	CDH5	Cadherin-5	27		1.468
17	P04186	CFB	Complement factor B	19		0.923
18	P06909	CFH	Complement factor H	45	1.234	
19	Q61129	CFI	Complement factor I	8		0.870
20	P12960	CNTN1	Contactin-1	59		1.214
21	Q61147	CP	Ceruloplasmin	54	1.116	
22	P09581	CSF1R	Macrophage colony-stimulating factor 1 receptor	5		1.178
23	P63037	DNAJA1	DnaJ homolog subfamily A member 1	26		0.258
**24**	**Q61508**	**ECM1**	**Extracellular matrix protein 1**	**18**	**0.767**	**0.846**
25	Q01279	EGFR	Epidermal growth factor receptor	46		1.137
26	P19221	F2	Prothrombin	26		0.972
27	Q9QXC1	FETUB	Fetuin-B	8		1.214
28	E9PV24	FGA	Fibrinogen alpha chain	30	1.435	
29	P11276	FN1	Fibronectin	145		1.008
30	P21614	GC	Vitamin D-binding protein	23		1.253
**31**	**P13020**	**GSN**	**Gelsolin**	**44**	**0.902**	**0.987**
32	P01898	H2-Q10	H-2 class I, Q10 alpha chain	11		1.212
33	P01942	HBA	Hemoglobin subunit alpha	18	1.467	
**34**	**P02088**	**HBB-B1**	**Hemoglobin subunit beta-1**	**19**	**1.460**	**1.309**
35	Q61646	HP	Haptoglobin	14	3.311	
36	Q91X72	HPX	Hemopexin	24	1.328	
37	P06330	IG HEAVY C	Ig heavy chain V region AC38 205.12	3	1.116	
**38**	**P01864**	**IGG**	**Ig gamma-2A chain C region secreted form**	**4**	**1.310**	**1.849**
**39**	**P01867**	**IGH-3**	**Ig gamma-2B chain C region**	**9**	**1.821**	**2.659**
**40**	**P01872**	**IGHM**	**Ig mu chain C region**	**16**	**1.585**	**1.696**
**41**	**P01837**	**IGK**	**Ig kappa chain C region**	**5**	**1.242**	**1.514**
42	Q61702	ITIH1	Inter-alpha-trypsin inhibitor heavy chain H1	17	1.174	
43	Q61703	ITIH2	Inter-alpha-trypsin inhibitor heavy chain H2	33		0.907
**44**	**Q61704**	**ITIH3**	**Inter-alpha-trypsin inhibitor heavy chain H3**	**14**	**1.518**	**0.937**
**45**	**A6X935**	**ITIH4**	**Inter alpha-trypsin inhibitor, heavy chain 4**	**23**	**1.632**	**0.993**
46	P04104	KRT1	Keratin, type II cytoskeletal 1	10		0.533
47	P08730	KRT13	Keratin, type I cytoskeletal 13	30		0.933
48	Q6NXH9	KRT73	Keratin, type II cytoskeletal 73	10		0.588
49	Q61233	LCP1	Plastin-2	44	0.672	
**50**	**P42703**	**LIFR**	**Leukemia inhibitory factor receptor**	**25**	**0.736**	**0.712**
51	Q80XG9	LRRTM4	Leucine-rich repeat transmembrane neuronal protein 4	9		0.751
52	P51885	LUM	Lumican	12	0.922	
53	O09159	MAN2B1	Lysosomal alpha-mannosidase	40	0.800	
54	P04247	MB	Myoglobin	13		1.354
55	Q99KE1	ME2	NAD-dependent malic enzyme, mitochondrial	37		0.751
56	P28665	MUG1	Murinoglobulin-1	63	0.877	
57	P11589	MUP2	Major urinary protein 2	8	0.896	
58	P97863	NFIB	Nuclear factor 1 B-type	21		0.358
59	O89084	PDE4A	cAMP- 3′,5′-cyclic phosphodiesterase 4A	21		0.718
60	P52480	PKM	Pyruvate kinase PKM	60	0.605	
61	Q60963	PLA2G7	Platelet-activating factor acetylhydrolase	18		1.110
62	P20918	PLG	Plasminogen	39		1.073
**63**	**P55065**	**PLTP**	**Phospholipid transfer protein**	**12**	**1.102**	**1.420**
64	P52430	PON1	Serum paraoxonase/arylesterase 1	14		1.137
65	Q62009	POSTN	Periostin	39		1.183
**66**	**Q61171**	**PRDX2**	**Peroxiredoxin-2**	**17**	**1.303**	**1.280**
67	Q61838	PZP	Pregnancy zone protein	70		1.052
68	P07758	SERPINA1A	Alpha-1-antitrypsin 1-1	18		0.963
69	P22599	SERPINA1B	Alpha-1-antitrypsin 1-2	6	0.824	
**70**	**Q00897**	**SERPINA1D**	**Alpha-1-antitrypsin 1-4**	**8**	**0.835**	**0.832**
71	P07759	SERPINA3K	Serine protease inhibitor A3K	14		1.076
72	Q91WP6	SERPINA3N	Serine protease inhibitor A3N	17		1.503
73	P32261	SERPINC1	Antithrombin-III	26		0.926
74	P49182	SERPIND1	Heparin cofactor 2	9		0.994
75	P97298	SERPINF1	Pigment epithelium-derived factor	18	0.946	
76	Q61247	SERPINF2	Alpha-2-antiplasmin	6		0.841
77	P97290	SERPING1	Plasma protease C1 inhibitor	12	1.149	
78	P70441	SLC9A3R1	Na(+)/H(+) exchange regulatory cofactor	29		1.156
79	P35441	THBS1	Thrombospondin-1	63		1.233
80	Q9Z1T2	THBS4	Thrombospondin-4	23		1.220
81	P07309	TTR	Transthyretin	10		1.242
**82**	**P29788**	**VTN**	**Vitronectin**	**10**	**1.168**	**1.042**

*The UniProt protein identifiers (ID), protein IPA code, full name, and fold changes (FC) of significantly differentially expressed proteins (q-value < 0.05) following local heart irradiation at 8 or 16 Gy are shown. Cells without any value mean that the protein did not pass the selection criteria in the proteomics analysis (q-value < 0.05, protein identification with at least two unique peptides). The shared proteins are in bold*.

**Figure 1:**
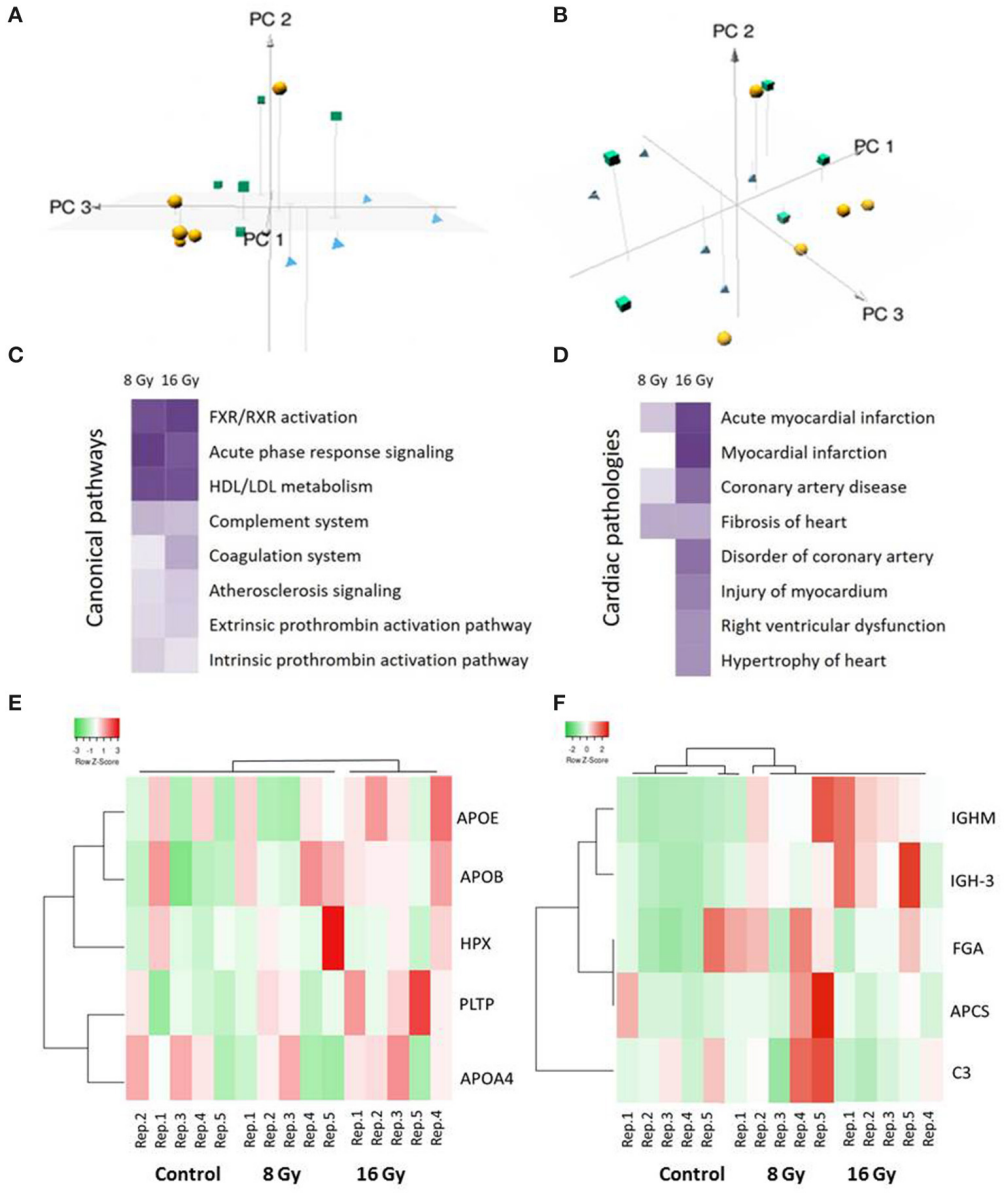
Multivariate, pathway, and cardiotoxicity analyses of the significantly differentially expressed serum proteins after local heart irradiation using 0 (control), 8, or 16 Gy. The principal component analysis (PCA) performed on normalized intensities of all proteins resulted in PC1, PC2, and PC3 as follows: PC1 15.9%, PC2 15.1%, and PC3 12.3%. The control samples are represented as yellow balls, the samples exposed to 8 Gy in green cubes, and the 16 Gy treated samples in blue pyramids **(A,B)**. A dose-dependent alteration is observed in the pathways involved in the inflammation and lipid metabolism **(C)**. Several proteins were identified associated with different heart pathologies **(D)**. The pathway and cardiotoxicity scores are displayed using a purple color gradient; the darker the color, the higher the scores and, thereby, statistical significance. The score is the negative log of the *p*-value derived from the Fisher's Exact test. By default, the rows (pathways) with the highest total scores across the set of observations are sorted to the top. The analysis was performed using Ingenuity Pathway Analysis (IPA) (Qiagen Inc., https://www.qiagenbioinformatics.com/products/ingenuity-pathway-analysis). The heat maps show hierarchical clustering (complete linkage, Spearman ranked correlation) of significantly deregulated proteins belonging to the high-density lipoprotein (HDL)/low-density lipoprotein (LDL) metabolism **(E)** and acute phase response **(F)** pathways in the control and irradiated samples. The green bars indicate downregulation and the red bars upregulation. The analysis was performed by the Heatmapper web server (http://www.heatmapper.ca/) (31). Detailed information of the proteomics features and individual samples is given in Supplementary Table 1.

To assess the global variation in the samples, a multivariate analysis was performed using three-dimensional principal component analysis (3D-PCA). The 3D-PCA, based on the normalized intensities of all serum proteins, showed a clustering among the different groups (PC1: 15.9%, PC2: 15.1%, and PC3: 12.3%) (Figures 1A,B). The 8- and 16-Gy treated samples were separated mainly on the PC2 axis, whereas the discrimination between the controls and 8-Gy treated samples was visible on the PC3 axis.

In particular, apolipoproteins, serpins, immunoglobulins, and inter-alpha-trypsin inhibitors were differentially regulated in the irradiated mice at both doses (Table 1). These shared proteins are mainly involved in the inflammatory response, and cholesterol and lipid metabolism. A detailed analysis of functional interactions and biological pathways based on differentially regulated proteins showed that acute phase response signaling, LXR/RXR cascade, cholesterol metabolism, coagulation system, and atherosclerosis signaling were the most affected pathways (Figure 1C and Supplementary Table 2). The differentially regulated proteins are associated with several heart pathologies such as infarction, hypertrophy, and fibrosis (Figure 1D and Supplementary Table 3). The analysis indicated a dose-dependent increase in the significance of the influenced pathways and in the cardiac pathologies.

Based on the list of canonical pathways (Figure 1C) the deregulated proteins belonging to two of the significantly affected pathways, namely, HDL/LDL metabolism and acute phase response signaling, were subjected to hierarchical clustering analysis (Figures 1E,F) (31). The heat map showed a clustering associated with the irradiation status of the groups.

**Figure 2:**
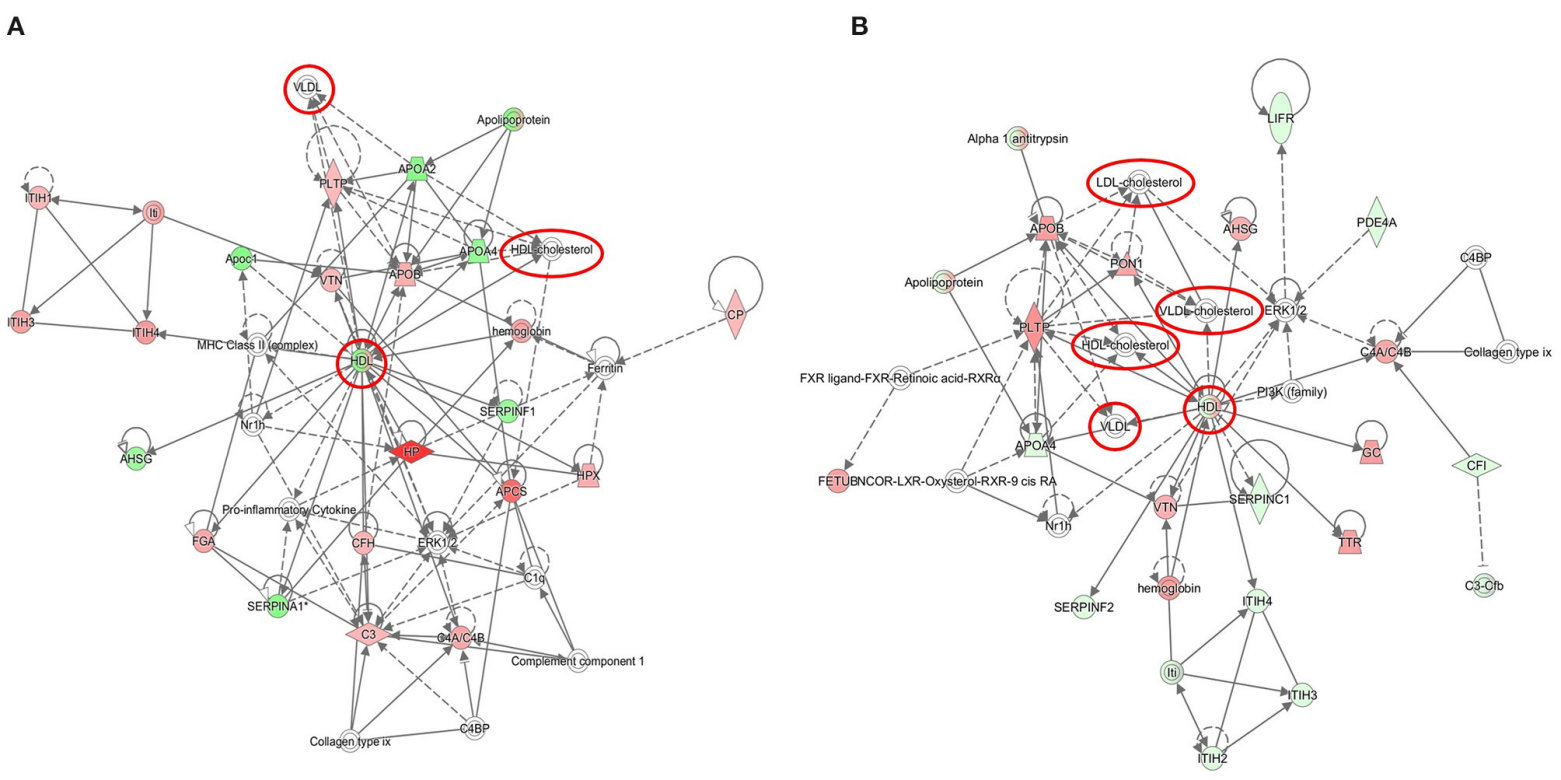
Graphical representation of cholesterol-associated networks based on radiation-induced alterations in the serum proteome. The protein clusters are shown at 8 Gy **(A)** and 16 Gy **(B)**. The upregulated proteins are marked in red and the downregulated in green. The nodes represent proteins connected with arrows; the solid arrows represent direct interactions and the dotted arrows indirect interactions. The cholesterol nodes are marked inside red circles and boxes. The IPA codes and corresponding full protein names are shown in Table 1 and in Supplementary Table 1. The analysis was performed using the Ingenuity Pathway Analysis (IPA) (https://www.qiagenbioinformatics.com/products/ingenuity-pathway-analysis).

The significantly deregulated proteins built a functional network associated with cholesterol metabolism and transport (Figure 2 and Supplementary Table 4). All deregulated proteins formed a tight cluster interacting with regulatory proteins of the inflammatory and acute phase response pathways.

**Figure 3:**
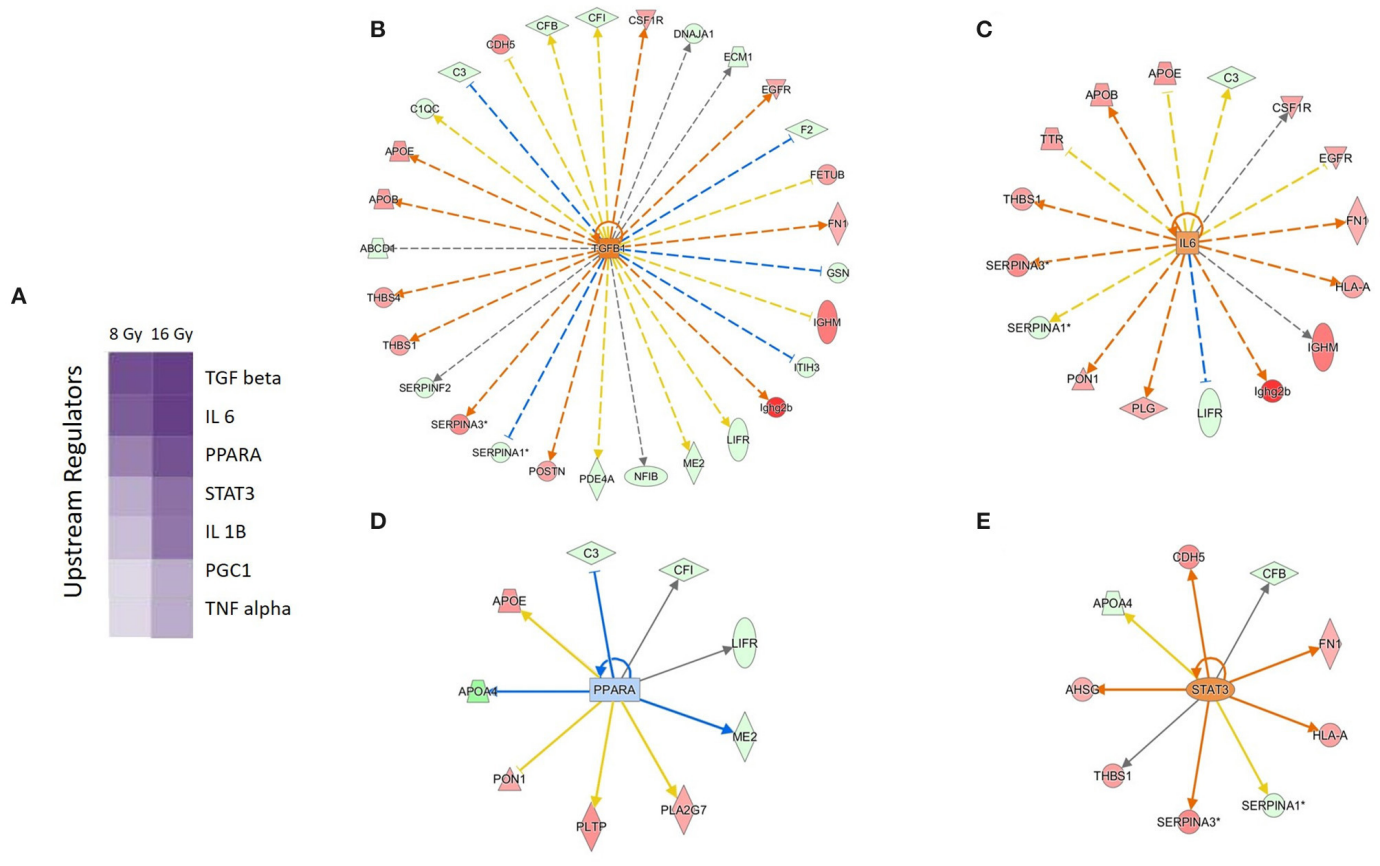
Predicted upstream regulators of the deregulated serum proteins. Predicted upstream regulators are displayed using a purple color gradient where the intensity of the purple color corresponds to statistical significance (the deeper the color, the higher the significance). The score is the negative log of the *p*-value derived from Fisher's exact test. By default, the rows (upstream regulators) with the highest total scores across the set of observations are sorted to the top **(A)**. The predicted upstream regulators and their activity status at 16 Gy are shown: TGF-β **(B)**, IL-6 **(C)**, PPARα **(D)**, and STAT3 **(E)**. The orange and the blue color of the nodes indicate activation and deactivation, respectively; the solid arrows represent direct interactions and the dotted arrows indirect interactions. The deregulated proteins forming the wheel around the nodes are marked in red (upregulation) and green (downregulation). The IPA codes and corresponding full protein names are shown in Table 1 and in Supplementary Table 1. The analysis was performed using Ingenuity Pathway Analysis (IPA) (https://www.qiagenbioinformatics.com/products/ingenuity-pathway-analysis).

The prediction analysis of the upstream regulators of the significantly deregulated proteins identified transcription factors involved in proinflammatory response (IL-6, TGF-β, and STAT3) and lipid metabolism (PPARα, PGC-1). The proinflammatory regulators were predicted to be activated, while PPARα was predicted to be inactivated (Figure 3 and Supplementary Table 5).

### Radiation-Induced Serum Inflammatory Markers

**Figure 4:**
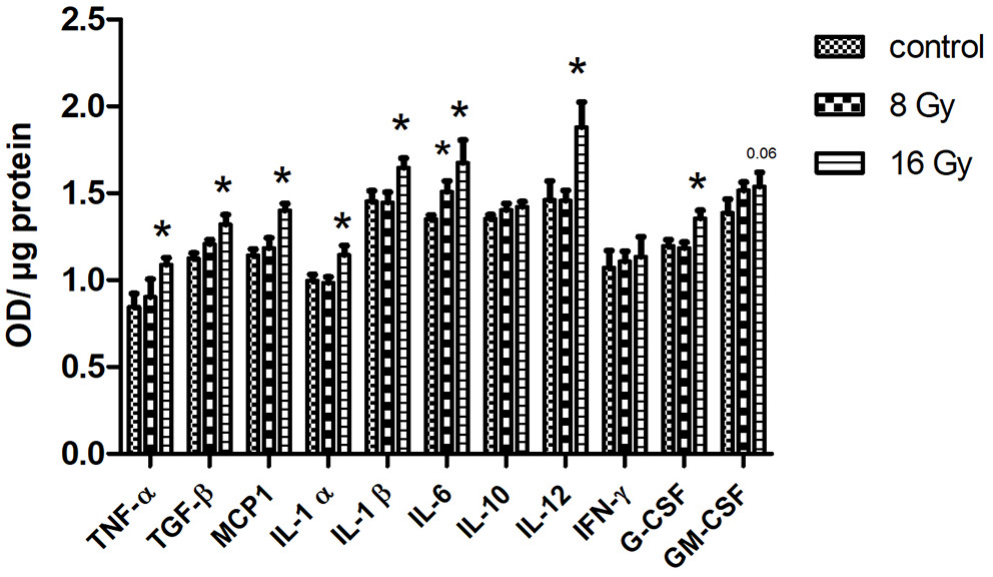
The ELISA analysis of serum cytokines. The level of cytokines was measured in 100 μg of serum in mice following 8 or 16 Gy local heart radiation using ELISA (*t*-test; *p < 0.05, mean with SEM, *n* = 5).

Since the inflammatory response was the main affected pathway in the serum proteome following local heart irradiation, the level of 11 different cytokines and inflammatory mediators was measured in serum using ELISA. At 8 Gy, only the level of IL-6 significantly increased. In contrast, following 16 Gy, the serum levels of TNF-α, TGF-β, MCP-1, IL-1 α and β, IL-6, IL-12, and G-CSF were significantly increased in comparison with controls (Figure 4). The level of IFN-γ, IL-10, and GM-CSF remained unchanged after irradiation.

### Radiation-Associated Changes in Serum Lipids

**Figure 5:**
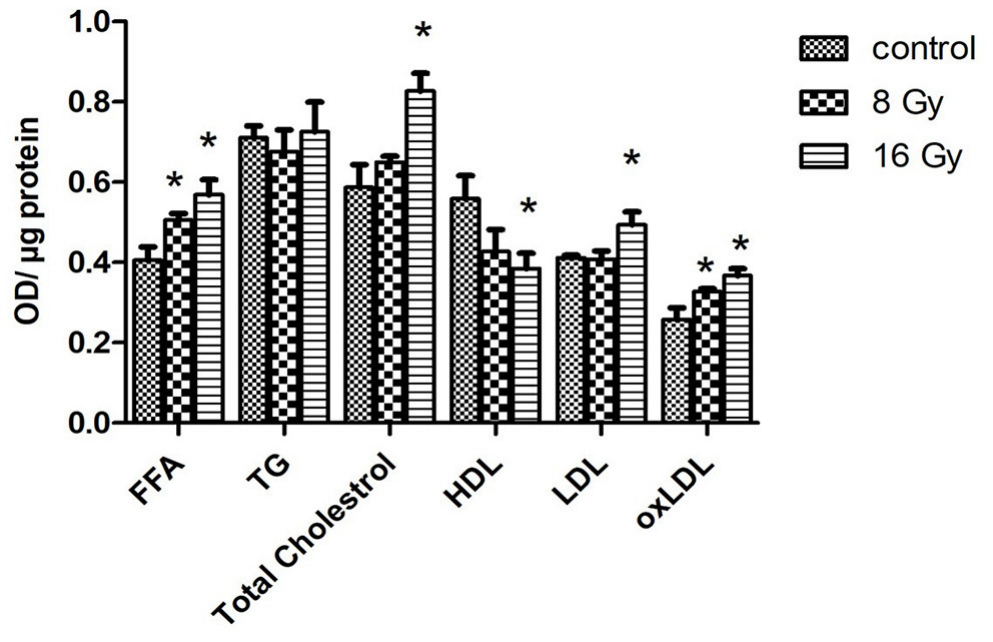
The ELISA analysis of serum lipid levels. The levels of free fatty acid (FFA), triglyceride (TG), total cholesterol, HDL, LDL, and oxidized low-density lipoprotein (oxLDL) were measured in 100 μg of serum of mice at 8- or 16-Gy local heart irradiation using ELISA (*t*-test; *p < 0.05, mean with SEM, *n* = 5).

The changes in the serum proteome indicated alterations in lipid metabolism. Therefore, the level of free fatty acid (FFA), total cholesterol, high-density lipoprotein (HDL), low-density lipoprotein (LDL), and triglyceride (TG) was measured in the serum of the control and irradiated mice. The level of FFA was increased at both radiation doses, while the levels of total cholesterol and LDL were increased only at 16 Gy. Similarly, the level of HDL was reduced only at 16 Gy (Figure 5). The level of TG remained unchanged in irradiated mice.

To examine the effect of oxidative stress on the level of serum lipids, the level of oxidized LDL (oxLDL) was analyzed. The analysis confirmed an enhanced level of oxLDL at both radiation doses (Figure 5).

## Discussion

The serum proteome is a reliable mirror of the individual's healthy and diseased states (33). In this study, we used global serum proteomics analysis as a starting point to predict radiation effects outside the target tissue. Applying a multivariate analysis on the data, in this case principal component analysis and hierarchical clustering, we could separate the control group from the irradiated groups. Although the analysis could even differentiate between the two irradiated groups based on the radiation dose, it also highlighted a panel of proteins being differentially expressed in both irradiated groups. This panel, rather than one single protein, can be considered as a radiation biomarker in the serum proteome.

This analysis also clearly showed that local heart irradiation is able to induce systemic inflammation and hypercholesterolemia in mice. These two responses are similar to those found in a multiomics study comparing atherogenic and dyslipidemic mice with wild-type mice and, more importantly, when comparing familial hypercholesterolemia patients with healthy controls (34).

The degree of this systemic inflammatory and dyslipidemic effect was dose-dependent and thereby presumably also related to the degree of the heart damage. The dose of 8 Gy was only partly able to induce similar proteome changes as the 16-Gy dose, and the proteome was, in general, altered to a lesser extent. Furthermore, the lower radiation dose was not able to alter the cytokine or lipid profile of the serum as strongly as the higher dose.

The pathological changes in the locally irradiated heart tissue of these mice have been described in our previous study (35) where we showed radiation-induced elevation of inactive phosphorylated PPARα and increased expression levels of proteins involved in SMAD-dependent and SMAD-independent TGF-β signaling. Furthermore, we showed enhanced levels of proteins involved in fibroblast to myofibroblast conversion and inflammation at 16 Gy. Some, but not all, of these protein expression changes were also present at 8 Gy (35). Histological examination in similarly treated C57BL/6J mice revealed a significant increase in epicardial thickness (8 and 16 Gy), enhanced levels of inflammatory cells, and iron-containing macrophages (16 Gy) after 20 weeks (36). These changes are in line with the alterations found in the serum of irradiated mice in this study.

We have shown previously that, particularly, cardiac endothelial cells respond to high-dose radiation by secreting proinflammatory cytokines *in vivo* and *in vitro* (19, 37–39). TNF-α that we found significantly elevated at the 16-Gy dose modulates the inflammatory response by activating the expression of IL-1 and IL-6 (40). These cytokines that also were upregulated in the serum of irradiated animals serve as significant predictors of cardiovascular disease (40, 41). In agreement with our data, elevated levels of IL-1 and IL-6 were found in patients after radiation therapy for lung cancer (42).

We found in this study changed levels in serum proteins involved in blood clotting in irradiated mice, indicating not only inflammatory but also thrombotic changes. Among these were several serpins, plasminogen, fibronectin, and fibrinogen. Fibrinogen is a serum adhesion molecule identified in individuals with a high risk for cardiovascular disorders (43). IL-1 and IL-6 positively influence the synthesis of fibrinogen (44, 45). Fibrinogens contribute to atherosclerotic plaque formation by inducing endothelial permeability and increase the probability for thrombus formation by enhancing the blood viscosity and platelet aggregation (44, 46, 47). In agreement with our results, previous studies show an induction of thrombotic responses in locally irradiated carotid and saphenous arteries and in the heart (48–50).

The proteomics data in this study predicted radiation-induced activation of TGF-β, and its upregulation in the serum was confirmed at 16 Gy using ELISA. TGF-β is a multifunctional cytokine regulating inflammation and fibrosis in the heart (51, 52). The consequences of cardiac fibrosis are severe including contractile dysfunction, deformation and remodeling of the cardiac structure, and heart failure (53). Enhanced levels of TGF-β mediate also radiation-induced cardiac fibrosis that is characterized by excess fibroblast proliferation and deposition of collagen fibers (36, 54). We have shown previously the activation of TGF-β signaling and induction of fibrosis in the mouse heart exposed to local high-dose radiation (16 Gy) (35, 55).

In contrast to the systemic inflammatory effect, this is, to the best of our knowledge, the first study to show that local heart irradiation has a profound effect on serum lipids. Enhanced levels of free fatty acids and total cholesterol that we find here, especially in the 16-Gy irradiated mice, are strong risk factors for cardiovascular disease (56, 57). Similarly, increased LDL and decreased HDL levels, particularly in combination, are associated with increased risk for cardiovascular disease in humans since, if long lasting, they are known to lead to hardening of the arteries and atherosclerosis (58). The enhancement of oxLDL serum level that we have observed already in a previous study (19) is a strong predictive marker for upcoming coronary heart disease events in healthy men and a potential risk factor for cardiovascular disease (59, 60). OxLDL is involved in the early progression of the atherosclerotic plaque formation including endothelial injury, increased levels of adhesion molecules, leukocyte recruitment and extravasation, and foam cell and thrombus formation (61). Moreover, it activates the inflammatory response and increases the production of cytokines (62).

The transcription factor PPARα that was predicted to be inactivated in irradiated animals, based on the serum proteome profiling, is the main regulator of lipid metabolism (63). Furthermore, it exerts anti-inflammatory effects in the vascular wall and, thereby, protects against initiation and progression of atherosclerosis (64). The PPARα protein is highly expressed in the heart but not excreted in the serum. We have shown previously that cardiac PPARα is inactivated after local heart irradiation in mice (28). More importantly, it was inactivated in a dose-dependent manner in the cardiac left ventricle of Mayak nuclear workers exposed to varying total body doses of external gamma radiation when compared with Mayak workers not exposed to irradiation (10). Both exposed and control workers were diagnosed and died of ischemic heart disease. These data indicate that, although deactivation of PPARα is a common feature in ischemic heart disease and has been observed in human heart failure patients (65–69), it is especially prominent in radiation-induced heart disease and, therefore, a radiation target in the heart. It is particularly interesting that this is reflected in the serum proteome and cytokine and lipid profiles.

Immunoglobulins G and M were significantly upregulated in the serum of irradiated mice. Increased levels of both immunoglobulins in blood have been associated with adverse cardiovascular events, particularly in dyslipidemic men, but the epidemiological data are contradictory (70–73). In contrast, we did not identify cardiac troponins that are immediate markers of cardiac damage in humans as in mice. In mice, cardiac TnI concentrations in serum peaked at 1 to 4 h and declined to baseline by 48–72 h after a single administration of isoproterenol (74). This rapid decline is probably the reason why we did not find it elevated in the mouse serum 20 weeks after local heart radiation. Nevertheless, cardiac troponins seem to stay downregulated in the cardiac tissue a long time after radiation exposure. We have shown previously a dose-dependent decrease in cTnT in the human left ventricle in the Mayak worker study (10) and cTnI in the locally irradiated mouse heart (8 and 16 Gy) (28) suggesting an early leakage of cardiac troponins to the serum after radiation-induced myofibril degradation.

All in all, the data presented here suggest that the serum proteins and lipids function as potential biomarkers of cardiac injury following heart high-dose radiation exposure. They confirm our previous findings in the heart proteome following high-dose irradiation suggesting radiation-associated activation of TGF-β but inactivation of PPARα (35, 56). Especially, PPARα has become an interesting therapeutic target due to its pleiotropic activity in controlling lipid metabolism and energy homeostasis, inhibiting inflammation, reducing oxidative stress and apoptosis, and ameliorating contractile function. However, the clinical trials using PPARα agonists have shown contradictory outcomes so far (75). We suggest that administering such agonists could be particularly beneficial in connection with radiation therapy for thoracic malignancies where the heart may receive considerable radiation doses leading to adverse cardiovascular events (76). Furthermore, the data from this serum study could be beneficial in identifying patients who may develop radiation-associated cardiac toxicity.

## Data Availability Statement

The datasets presented in this study can be found in online repositories. The names of the repository/repositories and accession number(s) can be found in the article/Supplementary Material.

## Ethics Statement

All animal experiments were approved and licensed under Bavarian federal law (Certificate No. AZ 55.2-1-54-2532-114-2014).

## Author Contributions

The study was designed by OA, VS, MA, GM, and ST. The irradiation was done by WS and VS. Serum collection was done by VS. The proteomics analysis was done by CT and OA. The ELISA experiments were performed by OA. The multivariate analysis was done by OA. OA and ST wrote the draft manuscript. All authors contributed to the revision of the manuscript, read it, discussed, and approved the final version.

## Conflict of Interest

The authors declare that the research was conducted in the absence of any commercial or financial relationships that could be construed as a potential conflict of interest.

## References

[1] Darby SC Ewertz M McGale P Bennet AM Blom-Goldman U Bronnum D. Risk of ischemic heart disease in women after radiotherapy for breast cancer. N Engl J Med. (2013) 368:987–98. 10.1056/NEJMoa1209825 23484825

[2] Swerdlow AJ Higgins CD Smith P Cunningham D Hancock BW Horwich A. Myocardial infarction mortality risk after treatment for Hodgkin disease: a collaborative British cohort study. J Natl Cancer Inst. (2007) 99:206–14. 10.1093/jnci/djk029 17284715

[3] Okwuosa TM Anzevino S Rao R. Cardiovascular disease in cancer survivors. Postgrad Med J. (2017) 93:82–90. 10.1136/postgradmedj-2016-134417 28123076

[4] Yusuf SW Venkatesulu BP Mahadevan LS Krishnan S. Radiation-induced cardiovascular disease: a clinical perspective. Front Cardiovasc Med. (2017) 4:66. 10.3389/fcvm.2017.00066 29124057 PMC5662579

[5] Demirci S Nam J Hubbs JL Nguyen T Marks LB. Radiation-induced cardiac toxicity after therapy for breast cancer: interaction between treatment era and follow-up duration. Int J Radiat Oncol Biol Phys. (2009) 73:980–7. 10.1016/j.ijrobp.2008.11.016 19251085

[6] Adams MJ Hardenbergh PH Constine LS Lipshultz SE. Radiation-associated cardiovascular disease. Crit Rev Oncol Hematol. (2003) 45:55–75. 10.1016/S1040-8428(01)00227-X 12482572

[7] Tapio S. Pathology and biology of radiation-induced cardiac disease. J Radiat Res. (2016) 57:439–48. 10.1093/jrr/rrw064 27422929 PMC5045085

[8] Cuomo JR Sharma GK Conger PD Weintraub NL. Novel concepts in radiation-induced cardiovascular disease. World J Cardiol. (2016) 8:504–19. 10.4330/wjc.v8.i9.504 27721934 PMC5039353

[9] Boerma M Sridharan V Mao XW Nelson GA Cheema AK Koturbash I. Effects of ionizing radiation on the heart. Mutat Res. (2016) 770:319–27. 10.1016/j.mrrev.2016.07.003 27919338 PMC5144922

[10] Azimzadeh O Azizova T Merl-Pham J Subramanian V Bakshi MV Moseeva M. A dose-dependent perturbation in cardiac energy metabolism is linked to radiation-induced ischemic heart disease in Mayak nuclear workers. Oncotarget. (2017) 8:9067–78. 10.18632/oncotarget.10424 27391067 PMC5354715

[11] Azimzadeh O Azizova T Merl-Pham J Blutke A Moseeva M Zubkova O. Chronic occupational exposure to ionizing radiation induces alterations in the structure and metabolism of the heart: a proteomic analysis of human formalin-fixed paraffin-embedded (FFPE) cardiac tissue. Int J Mol Sci. (2020) 21:6832. 10.3390/ijms21186832 32957660 PMC7555548

[12] Sridharan V Aykin-Burns N Tripathi P Krager KJ Sharma SK Moros EG. Radiation-induced alterations in mitochondria of the rat heart. Radiat Res. (2014) 181:324–34. 10.1667/RR13452.1 24568130 PMC4029615

[13] Henri C Heinonen T Tardif JC. The role of biomarkers in decreasing risk of cardiac toxicity after cancer therapy. Biomark Cancer. (2016) 8:39–45. 10.4137/BIC.S31798 27257396 PMC4878717

[14] Tian S Hirshfield KM Jabbour SK Toppmeyer D Haffty BG Khan AJ. Serum biomarkers for the detection of cardiac toxicity after chemotherapy and radiation therapy in breast cancer patients. Front Oncol. (2014) 4:277. 10.3389/fonc.2014.00277 25346912 PMC4191171

[15] Sharma S Jackson PG Makan J. Cardiac troponins. J Clin Pathol. (2004) 57:1025–6. 10.1136/jcp.2003.015420 15452153 PMC1770452

[16] Skyttä T Tuohinen S Boman E Virtanen V Raatikainen P Kellokumpu-Lehtinen PL. Troponin T-release associates with cardiac radiation doses during adjuvant left-sided breast cancer radiotherapy. Radiat Oncol. (2015) 10:141. 10.1186/s13014-015-0436-2 26159409 PMC4496940

[17] Wu AHB. Release of cardiac troponin from healthy and damaged myocardium. Front Lab Med. (2017) 1:144–50. 10.1016/j.flm.2017.09.003

[18] Canada JM Thomas GK Trankle CR Carbone S Billingsley H Van Tassell BW. Increased C-reactive protein is associated with the severity of thoracic radiotherapy-induced cardiomyopathy. Cardiooncology. (2020) 6:2. 10.1186/s40959-020-0058-1 32154028 PMC7048115

[19] Azimzadeh O Sievert W Sarioglu H Merl-Pham J Yentrapalli R Bakshi MV. Integrative proteomics and targeted transcriptomics analyses in cardiac endothelial cells unravel mechanisms of long-term radiation-induced vascular dysfunction. J Proteome Res. (2015) 14:1203–19. 10.1021/pr501141b 25590149

[20] Gkantaifi A Papadopoulos C Spyropoulou D Toumpourleka M Iliadis G Kardamakis D. Breast radiotherapy and early adverse cardiac effects. the role of serum biomarkers and strain echocardiography. Anticancer Res. (2019) 39:1667–73. 10.21873/anticanres.13272 30952705

[21] Ananthan K Lyon AR. The role of biomarkers in cardio-oncology. J Cardiovasc Transl Res. (2020) 13:431–50. 10.1007/s12265-020-10042-3 32642841 PMC7360533

[22] Cesari M Penninx BW Newman AB Kritchevsky SB Nicklas BJ Sutton-Tyrrell K. Inflammatory markers and onset of cardiovascular events: results from the Health ABC study. Circulation. (2003) 108:2317–22. 10.1161/01.CIR.0000097109.90783.FC 14568895

[23] Ueland T Gullestad L Nymo SH Yndestad A Aukrust P Askevold ET. Inflammatory cytokines as biomarkers in heart failure. Clin Chim Acta. (2015) 443:71–7. 10.1016/j.cca.2014.09.001 25199849

[24] Unger K Li Y Yeh C Barac A Srichai MB Ballew EA. Plasma metabolite biomarkers predictive of radiation induced cardiotoxicity. Radiother Oncol. (2020) 152:133–45. 10.1016/j.radonc.2020.04.018 32360032 PMC7572465

[25] Pietrowska M Wlosowicz A Gawin M Widlak P. MS-based proteomic analysis of serum and plasma: problem of high abundant components and lights and shadows of albumin removal. Adv Exp Med Biol. (2019) 1073:57–76. 10.1007/978-3-030-12298-0_3 31236839

[26] Lin L Zheng J Yu Q Chen W Xing J Chen C. High throughput and accurate serum proteome profiling by integrated sample preparation technology and single-run data independent mass spectrometry analysis. J Proteomics. (2018) 174:9–16. 10.1016/j.jprot.2017.12.014 29278786

[27] Smith JG Gerszten RE. Emerging affinity-based proteomic technologies for large-scale plasma profiling in cardiovascular disease. Circulation. (2017) 135:1651–64. 10.1161/CIRCULATIONAHA.116.025446 28438806 PMC5555416

[28] Azimzadeh O Sievert W Sarioglu H Yentrapalli R Barjaktarovic Z Sriharshan A. PPAR Alpha: a novel radiation target in locally exposed mus musculus heart revealed by quantitative proteomics. J Proteome Res. (2013) 12:2700–14. 10.1021/pr400071g 23560462

[29] Sievert W Stangl S Steiger K Multhoff G. Improved overall survival of mice by reducing lung side effects after high-precision heart irradiation using a small animal radiation research platform. Int J Radiat Oncol Biol Phys. (2018) 101:671–9. 10.1016/j.ijrobp.2018.02.017 29680258

[30] Kramer A Green J Pollard J Jr Tugendreich S. Causal analysis approaches in Ingenuity Pathway Analysis. Bioinformatics. (2014) 30:523–30. 10.1093/bioinformatics/btt703 24336805 PMC3928520

[31] Babicki S Arndt D Marcu A Liang Y Grant JR Maciejewski A. Heatmapper: web-enabled heat mapping for all. Nucleic Acids Res. (2016) 44:W147–53. 10.1093/nar/gkw419 27190236 PMC4987948

[32] Perez-Riverol Y Csordas A Bai J Bernal-Llinares M Hewapathirana S Kundu DJ. The PRIDE database and related tools and resources in 2019: improving support for quantification data. Nucleic Acids Res. (2019) 47:D442–50. 10.1093/nar/gky1106 30395289 PMC6323896

[33] Geyer PE Kulak NA Pichler G Holdt LM Teupser D Mann M. Plasma proteome profiling to assess human health and disease. Cell Syst. (2016) 2:185–95. 10.1016/j.cels.2016.02.015 27135364

[34] Olkowicz M Czyzynska-Cichon I Szupryczynska N Kostogrys RB Kochan Z Debski J. Multi-omic signatures of atherogenic dyslipidaemia: pre-clinical target identification and validation in humans. J Transl Med. (2021) 19:6. 10.1186/s12967-020-02663-8 33407555 PMC7789501

[35] Subramanian V Borchard S Azimzadeh O Sievert W Merl-Pham J Mancuso M. PPARalpha Is necessary for radiation-induced activation of noncanonical tgfbeta signaling in the heart. J Proteome Res. (2018) 17:1677–89. 10.1021/acs.jproteome.8b00001 29560722

[36] Seemann I Gabriels K Visser NL Hoving S te Poele JA Pol JF. Irradiation induced modest changes in murine cardiac function despite progressive structural damage to the myocardium and microvasculature. Radiother Oncol. (2012) 103:143–50. 10.1016/j.radonc.2011.10.011 22112779

[37] Philipp J Azimzadeh O Subramanian V Merl-Pham J Lowe D Hladik D. Radiation-induced endothelial inflammation is transferred via the secretome to recipient cells in a STAT-mediated process. J Proteome Res. (2017) 16:3903–16. 10.1021/acs.jproteome.7b00536 28849662

[38] Philipp J Sievert W Azimzadeh O von Toerne C Metzger F Posch A. Data independent acquisition mass spectrometry of irradiated mouse lung endothelial cells reveals a STAT-associated inflammatory response. Int J Radiat Biol. (2020) 96:642–50. 10.1080/09553002.2020.1712492 31914348

[39] Sievert W Trott KR Azimzadeh O Tapio S Zitzelsberger H Multhoff G. Late proliferating and inflammatory effects on murine microvascular heart and lung endothelial cells after irradiation. Radiother Oncol. (2015) 117:376–81. 10.1016/j.radonc.2015.07.029 26233589

[40] Azzawi M Hasleton P. Tumour necrosis factor alpha and the cardiovascular system: its role in cardiac allograft rejection and heart disease. Cardiovasc Res. (1999) 43:850–9. 10.1016/S0008-6363(99)00138-8 10615412

[41] Zhang H Park Y Wu J Chen X Lee S Yang J. Role of TNF-alpha in vascular dysfunction. Clin Sci. (2009) 116:219–30. 10.1042/CS20080196 PMC2620341 19118493

[42] Lierova A Jelicova M Nemcova M Proksova M Pejchal J Zarybnicka L. Cytokines and radiation-induced pulmonary injuries. J Radiat Res. (2018) 59:709–53. 10.1093/jrr/rry067 30169853 PMC6251431

[43] de Moerloose P Boehlen F Neerman-Arbez M. Fibrinogen and the risk of thrombosis. Semin Thromb Hemost. (2010) 36:7–17. 10.1055/s-0030-1248720 20391292

[44] Lominadze D Dean WL Tyagi SC Roberts AM. Mechanisms of fibrinogen-induced microvascular dysfunction during cardiovascular disease. Acta Physiol. (2010) 198:1–13. 10.1111/j.1748-1716.2009.02037.x 19723026 PMC2803614

[45] Rokita H Neta R Sipe JD. Increased fibrinogen synthesis in mice during the acute phase response: co-operative interaction of interleukin 1, interleukin 6, and interleukin 1 receptor antagonist. Cytokine. (1993) 5:454–8. 10.1016/1043-4666(93)90035-4 8142600

[46] Appiah D Schreiner PJ MacLehose RF Folsom AR. Association of plasma gamma' fibrinogen with incident cardiovascular disease: the atherosclerosis risk in communities (ARIC) study. Arterioscler Thromb Vasc Biol. (2015) 35:2700–6. 10.1161/ATVBAHA.115.306284 26494231 PMC4662615

[47] Stec JJ Silbershatz H Tofler GH Matheney TH Sutherland P Lipinska I. Association of fibrinogen with cardiovascular risk factors and cardiovascular disease in the Framingham Offspring Population. Circulation. (2000) 102:1634–8. 10.1161/01.CIR.102.14.1634 11015340

[48] Patties I Haagen J Dörr W Hildebrandt G Glasow A. Late inflammatory and thrombotic changes in irradiated hearts of C57BL/6 wild-type and atherosclerosis-prone ApoE-deficient mice. Strahlenther Onkol. (2015) 191:172–9. 10.1007/s00066-014-0745-7 25200359

[49] Patties I Habelt B Rosin B Dörr W Hildebrandt G Glasow A. Late effects of local irradiation on the expression of inflammatory markers in the Arteria saphena of C57BL/6 wild-type and ApoE-knockout mice. Radiat Environ Biophys. (2014) 53:117–24. 10.1007/s00411-013-0492-7 24071970

[50] Hoving S Heeneman S Gijbels MJ Te Poele JA Visser N Cleutjens J. Irradiation induces different inflammatory and thrombotic responses in carotid arteries of wildtype C57BL/6J and atherosclerosis-prone ApoE(-/-) mice. Radiother Oncol. (2012) 105:365–70. 10.1016/j.radonc.2012.11.001 23245647

[51] Frangogiannis NG. The role of transforming growth factor (TGF)-beta in the infarcted myocardium. J Thorac Dis. (2017) 9:S52–63. 10.21037/jtd.2016.11.19 28446968 PMC5383562

[52] Hassan MO Duarte R Dix-Peek T Dickens C Naidoo S Vachiat A. Transforming growth factor-beta protects against inflammation-related atherosclerosis in South African CKD patients. Int J Nephrol. (2018) 2018:8702372. 10.1155/2018/8702372 29977619 PMC6011064

[53] Parichatikanond W Luangmonkong T Mangmool S Kurose H. Therapeutic targets for the treatment of cardiac fibrosis and cancer: focusing on TGF-β signaling. Front Cardiovasc Med. (2020) 7:34. 10.3389/fcvm.2020.00034 32211422 PMC7075814

[54] Sun W Ni X Sun S Cai L Yu J Wang J. Adipose-derived stem cells alleviate radiation-induced muscular fibrosis by suppressing the expression of TGF-beta1. Stem Cells Int. (2016) 2016:5638204. 10.1155/2016/5638204 26649050 PMC4663335

[55] Subramanian V Seemann I Merl-Pham J Hauck SM Stewart FA Atkinson MJ. The role of TGF Beta and PPAR alpha signalling pathways in radiation response of locally exposed heart: integrated global transcriptomics and proteomics analysis. J Proteome Res. (2016) 16:307–18. 10.1021/acs.jproteome.6b00795 27805817

[56] Pilz S März W. Free fatty acids as a cardiovascular risk factor. Clin Chem Lab Med. (2008) 46:429–34. 10.1515/CCLM.2008.118 18605928

[57] Peters SA Singhateh Y Mackay D Huxley RR Woodward M. Total cholesterol as a risk factor for coronary heart disease and stroke in women compared with men: a systematic review and meta-analysis. Atherosclerosis. (2016) 248:123–31. 10.1016/j.atherosclerosis.2016.03.016 27016614

[58] Bhatnagar D Soran H Durrington PN. Hypercholesterolaemia and its management. Bmj. (2008) 337:a993. 10.1136/bmj.a993 18719012

[59] Stocker R Keaney JF Jr. Role of oxidative modifications in atherosclerosis. Physiol Rev. (2004) 84:1381–478. 10.1152/physrev.00047.2003 15383655

[60] Meisinger C Baumert J Khuseyinova N Loewel H Koenig W. Plasma oxidized low-density lipoprotein, a strong predictor for acute coronary heart disease events in apparently healthy, middle-aged men from the general population. Circulation. (2005) 112:651–7. 10.1161/CIRCULATIONAHA.104.529297 16043640

[61] Steinberg D. Lewis A. Conner memorial lecture. Oxidative modification of LDL and atherogenesis. Circulation. (1997) 95:1062–71. 10.1161/01.CIR.95.4.1062 9054771

[62] Rhoads JP Major AS. How oxidized low-density lipoprotein activates inflammatory responses. Crit Rev Immunol. (2018) 38:333–42. 10.1615/CritRevImmunol.2018026483 30806246 PMC6527110

[63] Gervois P Torra IP Fruchart JC Staels B. Regulation of lipid and lipoprotein metabolism by PPAR activators. Clin Chem Lab Med. (2000) 38:3–11. 10.1515/CCLM.2000.002 10774955

[64] Zandbergen F Plutzky J. PPARalpha in atherosclerosis and inflammation. Biochim Biophys Acta. (2007) 1771:972–82. 10.1016/j.bbalip.2007.04.021 17631413 PMC2083576

[65] Barger PM Kelly DP. PPAR signaling in the control of cardiac energy metabolism. Trends Cardiovasc Med. (2000) 10:238–45. 10.1016/S1050-1738(00)00077-3 11282301

[66] Sack MN Rader TA Park S Bastin J McCune SA Kelly DP. Fatty acid oxidation enzyme gene expression is downregulated in the failing heart. Circulation. (1996) 94:2837–42. 10.1161/01.CIR.94.11.2837 8941110

[67] Remondino A Rosenblatt-Velin N Montessuit C Tardy I Papageorgiou I Dorsaz PA. Altered expression of proteins of metabolic regulation during remodeling of the left ventricle after myocardial infarction. J Mol Cell Cardiol. (2000) 32:2025–34. 10.1006/jmcc.2000.1234 11040106

[68] Rosenblatt-Velin N Montessuit C Papageorgiou I Terrand J Lerch R. Postinfarction heart failure in rats is associated with upregulation of GLUT-1 and downregulation of genes of fatty acid metabolism. Cardiovasc Res. (2001) 52:407–16. 10.1016/S0008-6363(01)00393-5 11738057

[69] Dewald O Sharma S Adrogue J Salazar R Duerr GD Crapo JD. Downregulation of peroxisome proliferator-activated receptor-alpha gene expression in a mouse model of ischemic cardiomyopathy is dependent on reactive oxygen species and prevents lipotoxicity. Circulation. (2005) 112:407–15. 10.1161/CIRCULATIONAHA.105.536318 16009788

[70] Cristea A Rus H Niculescu F Bedeleanu D Vlaicu R. Characterization of circulating immune complexes in heart disease. Immunol Lett. (1986) 13:45–9. 10.1016/0165-2478(86)90124-0 3530991

[71] Kovanen PT Mänttäri M Palosuo T Manninen V Aho K. Prediction of myocardial infarction in dyslipidemic men by elevated levels of immunoglobulin classes A, E, and G, but not M. Arch Intern Med. (1998) 158:1434–9. 10.1001/archinte.158.13.1434 9665352

[72] Ravandi A Boekholdt SM Mallat Z Talmud PJ Kastelein JJ Wareham NJ. Relationship of IgG and IgM autoantibodies and immune complexes to oxidized LDL with markers of oxidation and inflammation and cardiovascular events: results from the EPIC-Norfolk Study. J Lipid Res. (2011) 52:1829–36. 10.1194/jlr.M015776 21821825 PMC3173004

[73] Khamis RY Hughes AD Caga-Anan M Chang CL Boyle JJ Kojima C. High serum immunoglobulin g and m levels predict freedom from adverse cardiovascular events in hypertension: a nested case-control substudy of the anglo-scandinavian cardiac outcomes trial. EBioMedicine. (2016) 9:372–80. 10.1016/j.ebiom.2016.06.012 27333022 PMC4972545

[74] Engle SK Jordan WH Pritt ML Chiang AY Davis MA Zimmermann JL. Qualification of cardiac troponin I concentration in mouse serum using isoproterenol and implementation in pharmacology studies to accelerate drug development. Toxicol Pathol. (2009) 37:617–28. 10.1177/0192623309339502 19549929

[75] Li S Yang B Du Y Lin Y Liu J Huang S. Targeting PPARα for the treatment and understanding of cardiovascular diseases. Cell Physiol Biochem. (2018) 51:2760–75. 10.1159/000495969 30562729

[76] Tapio S Little MP Kaiser JC Impens N Hamada N Georgakilas AG. Ionizing radiation-induced circulatory and metabolic diseases. Environ Int. (2021) 146:106235. 10.1016/j.envint.2020.106235 33157375 PMC10686049

